# Coincidence of diabetes mellitus and hypertension in a semi-urban Cameroonian population: a cross-sectional study

**DOI:** 10.1186/1471-2458-14-696

**Published:** 2014-07-08

**Authors:** Jean-Claude Katte, Anastase Dzudie, Eugene Sobngwi, Eta N Mbong, Gerard Tama Fetse, Charles Kouam Kouam, Andre-Pascal Kengne

**Affiliations:** 1 Regional Hospital of Bafoussam, Ministry of Public Health, Bafoussam, Cameroon; 2 Douala General Hospital and Faculty of Health Sciences, University of Buea, Buea, Cameroon; 3 Department of Medicine, Faculty of Health Sciences, University of Cape Town, Cape Town, South Africa; 4 Department of Internal Medicine and Subspecialties, Faculty of Medicine and Biomedical Sciences, University of Yaounde I, Yaounde, Cameroon; 5 Ministry of Public Health, Yaounde, Cameroon; 6 Non-Communicable Diseases Research Unit, South African Medical Research Council, PO Box 19070, Tygerberg, Cape Town 7505, South Africa

**Keywords:** Hypertension, Diabetes mellitus, Coincidence, Prevalence, Sub-Saharan Africa, Cameroon

## Abstract

**Background:**

Hypertension and diabetes mellitus are increasingly common in population within Africa. We determined the rate of coincident diabetes and hypertension and assessed the levels of co-awareness, treatment and control in a semi-urban population in Cameroon.

**Methods:**

A total of 1702 adults (967 women) self-selected from the community were consecutively recruited in Bafoussam (West region of Cameroon) during November 2012. Existing diabetes and hypertension and treatments were investigated and blood pressure and fasting blood glucose measured. Multinomial logistic regressions models were used to investigate the determinants of prevalent diabetes and hypertension.

**Results:**

Age-standardized prevalence rates (95% confidence intervals) men vs. women were 40.4% (34.7 to 46.1) and 23.8% (20.4 to 27.2) for hypertension alone; 3.3% (1.5 to 5.1) and 5.6% (3.5 to 7.7) for diabetes alone; and 3.9% (2.6 to 5.2) and 5.0% (3.5 to 6.5) for hypertension and diabetes. The age-standardized awareness, treatment and control rates for hypertension alone were 6.5%, 86.4% and 37.2% for men, and 24.3%, 52.1% and 51.6% in women. Equivalent figures for diabetes alone were 35.4%, 65.6% and 23.1% in men and 26.4%, 75.5% and 33.7% in women; and those for hypertension and diabetes were 86.6%, 3.3% and 0% in men, and 74.7%, 22.6% and 0% in women. Sex, age and adiposity were the main determinants of the three conditions.

**Conclusions:**

Coincident diabetes and hypertension is as high as diabetes alone in this population, driven by sex, age and adiposity. Awareness, treatment and control remain unacceptably low.

## Background

The prevalence of type 2 diabetes mellitus is increasing worldwide, and it is projected that the total number of people with diabetes will rise from 366 million in 2011 to 552 million by 2030 [1]. Hypertension is also a major public health challenge with an estimated global number of 972 million adults with the condition in 2000. This number is predicted to increase by 60% by 2025 [2,3]. Hypertension affects up to 70% of individuals with diabetes and is approximately twice as common in individuals with diabetes as in those without [4].

Diabetes mellitus and hypertension are among the commonest non-communicable diseases in both developed and developing countries [5]. The coexistence of diabetes and hypertension in the same individual varies across different ethnic, racial, and social groups [6]. Diabetes is an independent risk factor for cardiovascular disease, and the risk is markedly increased in the presence of hypertension. Furthermore, the overlap between diabetes and hypertension in the same individuals confers a greater risk of all target organ damages, general disability and premature mortality [7]. Moreover, the financial burden of the two conditions to patients, family and healthcare system is huge, particularly for the fragile developing economies [8]. High prevalence of hypertension, and more recently diabetes also, have been reported across developing countries in Africa, with people residing in urban settings being more affected [9,10]. There is also compelling evidence that more than half of individuals with each of the conditions at the community level are unaware of their status [11,12]. Furthermore, the percentages of patients on treatment and controlled to target levels are substantially low. Available community based reports have largely examined hypertension and diabetes separately, while the two conditions often coexist, share antecedents as well as barriers to detection, treatment and control. Community based details on the co-occurrence of hypertension and diabetes in Africa are therefore needed to improve the development and delivery of unified strategies to limit their harmful effects on the health of populations.

The present study aimed to determine the prevalence of coincident diabetes mellitus and hypertension and assess the levels of co-awareness, treatment and control in a semi urban population in Cameroon.

## Methods

### Study setting and population

This was a cross-sectional population-based study conducted in Bafoussam, the administrative Capital of the West Region of Cameroon. Bafoussam is a semi-urban city undergoing rapid population expansion and socio-economic changes. According to the year 2008 estimates [13,14], Bafoussam had a population of about 347,517 inhabitants. The current study was based on a self-selected sample. Between the 1^st^ to the 10^th^ of November 2012, daily announcements were made through two different radio networks in the town, inviting all interested and willing adults aged above 18 years to report to the five screening sites distributed across the city, for screening. Participants who reported to the screening sites were consecutively enrolled after they had signed an informed consent form. Participants were recruited from 12 to 14 November 2012, and the study was approved by the Cameroon National Ethics Committee.

### Data collection

Data collection was done simultaneously at the five screening sites by trained medical personnel during a face-to–face interview, using standardized questionnaire. Demographic characteristics, behavior profile, history of diabetes and hypertension were recorded. The blood pressure (BP) was then measured using a standardized protocol with the participant in a seated position, and after at least 10 minutes of rest. Three serial measurements, taken 1 minute apart, were obtained using automated sphygmomanometers (OMRON M2 Basic, OMRON HEALTHCARE Co Ltd, Kyoto, Japan). The average of the 2^nd^ and 3^rd^ measurement was used in all analysis. Weight, height and waist circumference (WC) were measured using standard procedures and equipment according to the World Health Organization guidelines [15]. Weight was measured to the nearest 0.5 kg and the height to the nearest 0.5 cm. Body mass index (BMI in kg/m^2^) was calculated as weight (kg) / [height (m) × height (m)]. Waist circumference (WC) was measured on the horizontal plane midway between the lower rib margin and the iliac crest with a measuring tape [15]. Fasting capillary blood glucose (FCG) was measured using a glucose meter (SD CodeFree blood glucose meter) based on the glucose oxidase enzyme method, after an overnight fast of at less 8 hours.

### Definitions

Hypertension was defined as a systolic (SBP) ≥140 mmHg or a diastolic blood pressure (DBP) ≥90 mmHg or on BP-lowering medication(s) during the past two weeks [16]. Participants with hypertension were considered to be aware of their status if they answered ‘yes’ to the question ‘Have you ever been told by a doctor or health professional that you had hypertension?’. Treatment of high BP was defined by the use of BP-lowering medications. Diabetes was defined as Fasting capillary glucose ≥126 mg/dL or previous diagnosis and on drug treatments (insulin or oral agents) during the past two weeks. Diagnosed diabetes was defined as those who were diagnosed by medical doctors or other health professionals and were on drug treatment in the past two weeks. Individuals who reported receiving anti-hyperglycaemic drugs in the past two weeks were considered as being on treatment. Individuals on diabetes treatment were considered as being controlled if their FCG was less than 100 mg/dL. Control target for blood pressure was based on SBP <140 mmHg and DBP <90 mmHg. The International Diabetes Federation (IDF) criteria [17] for African populations for obesity was used at cut-off point of BMI ≥30 kg/m2 and abdominal obesity, defined as WC ≥94 cm for men and ≥ 80 cm for women. People were considered to be physically active if they reported doing at least three sessions per week of physical activity of at less 30 minutes duration each. Moderate alcohol consumption was considered for any intake up to seven standard drinks per week for women and fourteen standard drinks per week for a man; with a standard drink defined as a bottle of beer or a glass of wine. Any consumption above was considered to be excessive.

### Statistical methods

Data analysis was carried out using the Statistical Package for Social Sciences v.17 for Windows (SPSS Inc., Chicago, USA). Results are summarized as count and percentages for qualitative variables and mean and standard deviation (SD) for quantitative variables. Individuals were categorized into four groups: no diabetes and no hypertension, diabetes only, hypertension only, and those with coexistence of diabetes and hypertension. Age-standardized prevalence was calculated using the Cameroon National population’s age structure in 2010 as the standard population [14], and direct standardization methods [18]. Comparison of groups was done using Chi-square tests and equivalents for qualitative variables, and Student t-test and analysis of the variance (ANOVA) for quantitative variables. Multinomial logistic regressions analyses were used to investigate potential determinants of hypertension alone, diabetes alone and the combination of both, in age and sex adjusted analysis and after further adjustment for potential confounders, always using participants with none of the conditions as a referent. Candidate predictors comprised age (categorical), sex, education, BMI (categorical), abdominal obesity, smoking, alcohol consumption and physical activity. A p-value <0.05 indicated statistically significant results.

## Results

### General characteristics of our study population

The overall profile of the 1702 participants (967 women; 56.8%) who reported for screening in the five screening sites is summarized in Table 1. Compared with men, women had a similar age (45 vs. 46 years, p = 0.35), similar waist girth 92 (SD = 44) vs. 94 (41) cm (p = 0.25), but higher BMI, 26.4 (4.5) vs. 29.3 (6.5) kg/m2 (p < 0.001). Men were more likely to be either smokers or excessive alcohol drinkers and more physically active (all p < 0.001). Mean systolic and diastolic blood pressure values (SD) at the total population level (men vs. women) were 146 (26) vs. 137 (26) mm Hg (p < 0.001), and 84 (25) vs. 81 (14) mm Hg (both p = 0.001). Equivalents figures for fasting blood glucose were 101 (42) vs. 104 (38) mg/dL (p = 0.14).

**Table 1:** General Characteristics of the study population

**Characteristics**	**Total**	**Men**	**Women**	**p-value**
**N (%)**	1702 (100%)	735 (43.2%)	967 (56.8)	
**Mean age, years (SD)**	45.7 (14.0)	46.1 (13.9)	45.4 (14.0)	0.35
**Age groups, years**				0.25
*< 35 years, n (%)*	404 (23.7)	174 (23.7)	230 (23.8)	
*35 – 44, n (%)*	415 (24.4)	170 (23.1)	245 (25.3)	
*45 – 54, n (%)*	439 (25.8)	182 (24.8)	257 (26.6)	
*≥ 55, n (%)*	444 (26.1)	209 (28.4)	235 (24.3)	
**Mean BMI, kg/m**^**2 **^**(SD)**	28.1 (5.9)	26.4 (4.5)	29.3 (6.5)	<0.001
**Mean waist girth, cm (SD)**	93 (42)	92 (44)	94 (41)	0.25
**Mean SBP, mm Hg (SD)**	141 (26)	146 (26)	137 (26)	<0.001
**Mean DBP, mm Hg (SD)**	82 (19)	84 (25)	81 (14)	0.001
**Education level**				<0.001
*No level (1), n (%)*	153 (9.0)	23 (3.1)	130 (13.4)	
*Primary (2), n (%)*	452 (26.6)	179 (24.4)	273 (28.2)	
*Secondary (3), n (%)*	807 (47.4)	367 (49.9)	440 (45.5)	
*University (4), n (%)*	290 (17.0)	166 (22.6)	124 (12.8)	
**Ever smoking**				<0.001
*Yes, n (%)*	199 (11(7)	169 (23.0)	30 (3.1)	
*No, n (%)*	1503 (88.3)	566 (77.0)	937 (96.9)	
**Alcohol consumption**				<0.001
*No, n (%)*	515 (30.3)	130 (17.7)	385 (39.8)	
*Moderate, n (%)*	1080 (63.5)	508 (69.1)	572 (59.2)	
*Excessive, n (%)*	107(6.3)	97 (13.2)	10 (1.0)	
**Physical activity**				<0.001
*Yes, n (%)*	1071 (62.9)	542 (73.7)	529 (54.7)	
*No, n (%)*	631 (37.1)	193 (26.3)	438 (45.3)	
**Mean Glycemia, mg/dL**	103 (40)	101 (42)	104 (38)	0.14

BMI, body mass index; DBP, diastolic blood pressure; SBP, systolic blood pressure; SD, standard deviation.

### Prevalence of hypertension and diabetes

The age standardized prevalence men vs. women and the accompanying 95% confidence intervals were 40.4% (34.7 to 46.1) and 23.8% (20.4 to 27.2) for hypertension alone; 3.3% (1.5 to 5.1) and 5.6% (3.5 to 7.7) for diabetes alone; and 3.9% (2.6 to 5.2) and 5.0% (3.5 to 6.5) for hypertension and diabetes (Table 2). The crude prevalence of hypertension alone increased from 33.3% (men) and 13.4% (women) in the age group <35 years, up to 61.2% (men) and 51.9% (women) in the age group 55 years and above (both p < 0.001 for linear trend; Table 2). The crude prevalence of hypertension and diabetes increased from 1.1% (men) and 2.2% (women) in the age group <35 years up to 13.4% (men) and 16.2% (women) in the age group 55 years and above (both p < 0.001 for linear trends). However, the crude prevalence of diabetes alone tended to decrease with increasing age and in a linear fashion (both p < 0.001 for linear trend, Table 2).

**Table 2:** Prevalence, awareness, treatment and control of hypertension alone, diabetes alone and the combination of both

**Condition**	**Men**							**Women**						
	**Overall**	**Overall standardized**	**Age groups (years)**				**p-trend**	**Overall**	**Overall standardized**	**Age groups (years)**				**p-trend**
			**<35**	**35-44**	**44-55**	**≥55**				**<35**	**35-44**	**44-55**	**≥55**	
N	735		174	170	182	209		967		230	245	257	235	
**Hypertension alone**														
SBP/DBP ≥ 140/90 or treatment (n)	359		58	67	106	128		345		31	69	123	122	
Prevalence (%)	48.8	40.4 (34.7 to 46.1)	33.3	39.4	58.2	61.2	<0.001	35.7	23.8 (20.4 to 27.2)	13.5	28.2	47.9	51.9	<0.001
Among hypertensives (n)	53		1	2	22	28		94		7	17	36	34	
Awareness (%)	14.8	6.5 (3.9 to 9.1)	1.7	3.0	20.7	21.9	<0.001	38.4	24.3 (13.6 to 35.0)	22.6	24.6	29.3	27.9	0.49
Among aware hypertensives (n)	40		1	1	16	22		64		3	10	24	27	
Treatment (%)	74.5	86.4 (−34.1 to 206.9)	100	50.0	72.7	78.6	0.67	68.1	52.1 (21.0 to 83.2)	42.9	58.8	66.7	79.4	0.031
Among treated hypertensives	36		0	1	14	21		51		1	8	19	23	
Control (%)	90.0	37.2 (5.1 to 69.3)	0	100	87.5	95.4	0.024	79.7	51.6 (9.6 to 93.6)	33.3	80.0	79.2	85.2	0.13
**Diabetes alone**														
FBS > 126 mg/dl or treatment (n)	22		6	7	4	5		51		14	10	14	13	
Prevalence (%)	3.0	3.3 (1.5 to 5.1)	3.4	4.1	2.3	2.4	<0.001	5.3	5.6 (3.5 to 7.7)	6.1	4.1	5.4	5.5	<0.001
Among diabetics (n)	8		2	1	2	3		17		3	2	5	7	
Awareness (%)	36.4	35.4 (4.8 to 66.0)	33.3	14.3	50.0	60.0	0.23	33.3	26.4 (9.7 to 43.1)	21.4	20.0	35.7	53.8	0.06
Among aware diabetics (n)	6		1	1	2	2		14		2	2	4	6	
Treatment (%)	75.0	65.6 (−4.4 to 135.6)	50.0	100	100	66.7	0.750	82.3	75.5 (12.9 to 138.1)	66.7	100	80.0	85.7	0.65
Among treated diabetics (n)	4		0	0	2	2		10		0	2	4	4	
Control (%)	66.7	23.1 (0.4 to 45.8)	0	0	100	100	0.048	71.4	33.7 (8.5 to 58.9)	0	100	100	66.7	0.29
**Hypertension & diabetes**														
SBP/DBP ≥ 140/90 or BP treatment & FBS > 126 mg/dl or diabetes treatment (n)	51		2	8	13	28		78		5	13	22	38	
Prevalence (%)	6.9	3.9 (2.6 to 5.2)	1.1	4.7	7.1	13.4	<0.001	8.1	5.0 (3.5 to 6.5)	2.2	5.3	8.6	16.2	<0.001
Among diabetic hypertensives (n)	35		2	6	5	22		46		4	11	13	18	
Awareness (%)	68.6	86.6 (1.5 to 171.7)	100	75.0	38.5	78.6	0.73	59.0	74.7 (25.0 to 124.4)	80.0	84.6	59.1	47.4	0.016
Among aware diabetic hypertensives (n)	3		0	0	1	2		11		1	0	4	6	
Treatment (%)	8.6	3.3 (−1.3 to 7.9)	0	0	20.0	9.1	0.24	23.9	22.6 (−8.3 to 53.5)	25.0	0	30.8	33.3	0.46
Among treated diabetic hypertensives (n)	0		0	0	0	0		0		0	0	0	0	
Control (%)	0	0	NA	NA	0	0	NA	0	0	0	NA	0	0	NA

DBP, diastolic blood pressure; FBS, fasting blood glucose; NA, not applicable; SBP, systolic blood pressure.

### Awareness, treatment and control of hypertension and diabetes

The age-standardized awareness, treatment and control rates for hypertension alone were 6.5%, 86.4% and 37.2% for men, and 24.3%, 52.1% and 51.6% in women (Table 2). Equivalent figures for diabetes alone were 35.4%, 65.6% and 23.1% in men and 26.4%, 75.5% and 33.7% in women; and those for hypertension and diabetes were 86.6%, 3.3% and 0% in men, and 74.7%, 22.6% and 0% in women. The crude prevalence of awareness increased with increasing age for hypertension alone in men (p < 0.001 for linear trend), while that for hypertension and diabetes decreased with increasing age in women (p = 0.016). A borderline decreasing trend of treatment with increasing age was observed for diabetes alone in women (p = 0.06 for linear trend), otherwise, trend in treatment rates was generally non-significant (all p ≥ 0.23 for linear trend). Control rate among those with hypertension alone increased with increasing age both in men (p = 0.024 for linear trend) and women (p = 0.031).

### Predictors of hypertension and diabetes

In age and sex adjusted multinomial logistic regressions models and using participants with none of the conditions as a reference group, male sex was associated with odd ratios (95% confidence interval) of 1.73 (1.39-2.16) for hypertension alone, 0.70 (0.42-1.19) for diabetes alone and 1.08 (0.72-1.60). The risk of hypertension alone, or hypertension and diabetes steadily increased with increasing age, with an apparent multiplicative effect of age on the risk of each of the conditions. The increasing risk of diabetes alone with age was apparent only when comparing participants in the upper age stratum (≥55 years) with those in the lower stratum (<35 years), with an odd ratio of 2.35 (1.20-4.61). Abdominal obesity was associated with odd ratio (95% CI) of 1.48 (1.12-1.94) for hypertension alone, 2.23 (1.13-4.40) for diabetes alone and 2.20 (1.34-3.60) for diabetes and hypertension (Table 3). The odds of hypertension alone, or hypertension with diabetes increased across BMI categories, while a significantly higher odd of diabetes alone was observed only for obese vs. normal weight participants [OR 2.04 (95% CI 1.08-3.86)]. Moderate alcohol consumption was associated with reduced odd of diabetes and hypertension [OR 0.58 (95% CI 0.38-0.88)], however the overall effect of alcohol consumption on the risk of the three outcomes was non-significant (p = 0.11 for the likelihood ratio chi square test). In expanded multivariable model, only sex, age and BMI categories were associated with the risk of the outcomes. The odd ratios from the final model comprising the three predictors are shown in Table 4.

**Table 3:** Age and sex adjusted multinomial logistic regressions for determinants of hypertension alone, diabetes alone or the combination of both

**Variable**	**Category**	**None**	**Hypertension alone**		**Diabetes alone**		**Diabetes and hypertension**		**Likelihood ratio test (p-value)**
			**OR (95% CI)**	**p-value**	**OR (95% CI)**	**p-value**	**OR (95% CI)**	**p-value**	
**Sex**	*Men*	1.00 (reference)	1.73 (1.39-2.16)	<0.001	0.70 (0.42-1.19)	0.19	1.08 (0.72-1.60)	0.72	<0.001
**Age (years)**									<0.001
	*< 35 years*	1.00 (reference)	1.00 (reference)	NA	1.00 (reference)	NA	1.00 (reference)	NA	
	*35 – 44 years*	1.00 (reference)	1.86 (1.35-2.57)	<0.001	1.00 (0.51-1.96)	0.99	3.59 (1.50-8.60)	0.004	
	*45 – 54 years*	1.00 (reference)	4.88 (3.56-6.69)	<0.001	1.62 (0.83-3.15)	0.16	9.21 (4.00-21.23)	<0.001	
	*≥ 55 years*	1.00 (reference)	7.39 (5.32-10.28)	<0.001	2.35 (1.20-4.61)	0.013	24.70 (10.99-55.50)	<0.001	
**Education level**									0.82
	*None*	1.00 (reference)	1.00 (reference)	NA	1.00 (reference)	NA	1.00 (reference)	NA	
	*Primary*	1.00 (reference)	0.98 (0.62-1.55)	0.93	0.51 (0.21-1.26)	0.14	0.81 (0.42-1.55)	0.52	
	*Secondary*	1.00 (reference)	1.14 (0.71-1.83)	0.57	0.49 (0.19-1.24)	0.13	0.96 (0.48-1.90)	0.90	
	*University*	1.00 (reference)	1.02 (0.60-1.74)	0.93	0.61 (0.21-1.77)	0.37	0.80 (0.33-1.93)	0.80	
**Smoking**									0.13
	*Yes*	1.00 (reference)	0.73 (0.51-1.04)	0.08	1.57 (0.72-3.40)	0.25	0.97 (0.53-1.77)	0.92	
**Alcohol consumption**									0.11
	*No*	1.00 (reference)	1.00 (reference)	NA	1.00 (reference)	NA	1.00 (reference)	NA	
	*Moderate*	1.00 (reference)	0.88 (0.68-1.13)	0.31	0.85 (0.51-1.43)	0.55	0.58 (0.38-0.88)	0.010	
	*Excessive*	1.00 (reference)	0.87 (0.53-1.42)	0.58	0.22 (0.03-1.75)	0.15	0.39 (0.14-1.09)	0.074	
**Physical activity**	*Yes*	1.00 (reference)	1.01 (0.80-1.27)	0.93	0.74 (0.45-1.22)	0.24	1.26 (0.83-1.91)	0.27	0.40
**Body mass index (kg/m**^**2**^**)**									<0.001
	*<25 kg/m*^*2*^	1.00 (reference)	1.00 (reference)	NA	1.00 (reference)	NA	1.00 (reference)	NA	
	*25 – 29.9 kg/m*^*2*^	1.00 (reference)	1.73 (1.32-2.26)	<0.001	1.27 (0.68-2.36)	0.45	2.12 (1.26-3.57)	0.005	
	*≥30 kg/m*^*2*^	1.00 (reference)	2.31 (1.71-3.13)	<0.001	2.04 (1.08-3.86)	0.029	3.94 (2.27-6.82)	<0.001	
**Abdominal obesity**									
	*Yes*	1.00 (reference)	1.48 (1.12-1.94)	0.005	2.23 (1.13-4.40)	0.14	2.20 (1.34-3.60)	0.002	0.002

NA, not applicable; OR, odd ratio; 95% CI, 95% confidence interval.

**Table 4:** Multivariable adjusted multinomial logistic regressions for determinants of hypertension alone, diabetes alone or the combination of both

**Variable**	**Categories**	**None**	**Hypertension alone**		**Diabetes alone**		**Diabetes and hypertension**		**Likelihood ratio test (p-value)**
			**OR (95% CI)**	**p-value**	**OR (95% CI)**	**p-value**	**OR (95% CI)**	**p-value**	
**Sex**	*Men*	1.00 (reference)	2.04 (1.62-2.57)	<0.001	0.82 (0.48-1.41)	0.48	1.41 (0.93-2.14)	0.14	<0.001
**Age (years)**									<0.001
	*< 35 years*	1.00 (reference)	1.00 (reference)	NA	1.00 (reference)	NA	1.00 (reference)	NA	
	*35 – 44 years*	1.00 (reference)	1.65 (1.19-2.29)	0.002	0.90 (0.46-1.77)	0.75	2.95 (1.23-7.10)	0.016	
	*45 – 54 years*	1.00 (reference)	4.39 (3.19-6.04)	<0.001	1.48 (0.76-2.91)	0.25	7.81 (3.37-18.08)	<0.001	
	*≥ 55 years*	1.00 (reference)	7.69 (5.51-10.74)	<0.001	2.43 (1.23-4.77)	0.010	26.32 (11.66-59.40)	<0.001	
**Body mass index (kg/m**^**2**^**)**									<0.001
	*<25 kg/m*^*2*^	1.00 (reference)	1.00 (reference)	NA	1.00 (reference)	NA	1.00 (reference)	NA	
	*25 – 29.9 kg/m*^*2*^	1.00 (reference)	1.73 (1.32-2.26)	<0.001	1.27 (0.68-2.36)	0.45	2.12 (1.26-3.57)	0.005	
	*≥30 kg/m*^*2*^	1.00 (reference)	2.31 (1.71-3.13)	<0.001	2.04 (1.08-3.86)	0.029	3.94 (2.27-6.82)	<0.001	

NA, not applicable; OR, odd ratio; 95% CI, 95% confidence interval.

## Discussion

This study has revealed the co-occurrence of hypertension and diabetes to be at least as frequent as diabetes alone. The study has further confirmed the high prevalence of hypertension in this population. While the presence of the three conditions appeared to be linked with increasing age and adiposity, effects on the risk of diabetes alone were significant only at the upper tail of the distribution of both characteristics. Within strata of age or BMI, the magnitude of the risk of diabetes and hypertension always appeared to be higher than that for hypertension alone, while a multiplicative effect was evident across age strata. Over ¾ of participants with diabetes and hypertension were aware of their condition, while only about 1/3^rd^ of those with diabetes alone and less than ¼ of those with hypertension alone were aware of their condition. Less than one in every five patients aware of their hypertension and diabetes was on co-treatment for the two conditions, against 2/3^rd^ or more for those with either of the conditions. Control rates among patients co-treated for diabetes and hypertension was insignificant, and ranged from 1/4^th^ to about half among those treated for either of the conditions. However, some of these figures were based on small numbers, hence the instability of the estimates.

The coexistence of diabetes and hypertension in the clinical setting has been extensively reported and described as a “toxic combination” that increases risks of cardiovascular diseases, renal complications, and retinopathy [19,20]. Choukem et al. [21] reported that 66.7% of diabetic patients in a clinical setting in Cameroon exhibit hypertension which is about three times more frequent than the general population. Contrariwise, community studies focusing on the prevalence of this “toxic combination, have been less well reported in sub-Saharan Africa. Some randomized control trial studies have demonstrated that aggressive control of blood glucose and blood pressure could improve the CVD outcomes [22]. Therefore early detection and control of the coexisting of both diseases is primordial and key to the reduction of related morbi-mortality. Our study revealed that more than 50% of the participants with the coexistence of both conditions were aware of their status but with less than 30% of individuals with both conditions being treated. None of the individuals with both conditions who were treated was simultaneously controlled. These figures are similar to those reported elsewhere [23] and therefore emphasize the need for a more pro-active approach to hypertension and diabetes prevention, detection, treatment and control in this setting. Possible drivers of the low awareness, detection, treatment and control of hypertension and diabetes in Cameroon and which could apply to the current study, have been extensively discussed previously [24,25].

There are some limitations to this study. First is the self-selected nature of participants that may not necessarily be representative of the city population. We attempted to achieve this by using an almost universal mass media for sensitization. However, we cannot rule out the possibility that participants in the study could still differ from the general population by being more or less health conscious, and having less severe disease for example, which could affect some parameters in the study, including the accuracy of our prevalence estimates. Therefore, caution should be exercised when extrapolation our estimates to a broader population in the same setting. Secondly, this study might have underestimated the true prevalence of diabetes since only fasting glucose test was used, and given that the two-hour glucose tolerance test has been reported to be more accurate in the detection of undiagnosed diabetes. However the fasting glucose test was more feasible to perform in this population-based study. Secondly, the diagnosis of hypertension was based on BP measurements during one single encounter which does not coincide with the current recommendation of using two measurements recorded on three separate occasions [26]. This may have overestimated the prevalence of hypertension and underestimated the level of BP control. Nevertheless, our study was based on a larger sample of participants from area not traditionally extensively covered by major previous population-based surveys in Cameroon. We have further used standardized measurement procedures to collect data in an accurate and reproducible way, and used robust analytic methods to generate estimates which will facilitate comparisons with evidence from elsewhere. Lastly, we didn’t assess the nutritional status which is a well-known major confounding factor for both hypertension and diabetes mellitus; nor did we collect the individual level data to investigate the determinants of detection, treatment and control of the two conditions in our population.

## Conclusions

In conclusion, high prevalence rates of diabetes and hypertension were observed in this population of self-selected semi-urban subjects. Increased age and BMI were found to be important risk factors for all these disorders. Integrated prevention, detection and control programs targeting both conditions and other major risk factors for cardiovascular diseases can potentially help to reverse the observed trends. However, locally relevant effective components of such programs have yet to be developed and fully tested.

## Competing interest

The authors declare that they have no competing interests.

## Authors’ contribution

Study conception: JCK, ENM, APK. Data collection: JCK, ENM, GTF, CKK. Data analysis: JCK, APK. Manuscript drafting: JCK, AD, APK. Critical revision of the manuscript: ES, ENM, GTF, CKK. Approval of the submission to the Journal: all authors.

## Pre-publication history

The pre-publication history for this paper can be accessed here:

http://www.biomedcentral.com/1471-2458/14/696/prepub
